# 

**Published:** 1888-09

**Authors:** 


### TABLE OF CONTENTS.

ORIGINAL AND SELECTED ARTICLES.

Summer diarrhœa of infants, by P. L. Cortelyou, M D,, Marietta, Ga. ... 321
Dublin Obstetrics, by E. S. McKee, M.D.. Cincinnati, Ohio ... 324
Veratrum as a specific in treatment of puerperal eclampsia, by F M Rushing, M. D ... 326
The dram-grain plan of wri ing prescriptions, by C. H. Merrick, M.D., Washington Ter ... 328
Cocaine in surgery, by Dr Friedrich Haenel ... 330
Clinical Society of London—conclusions of the myxœdema committee ... 334
Diuretic, sedative and diaphoretic pill, by J. B. Johnson, M.D., Washington, D. C ... 336

ABSTRACTS AND GLEANINGS.

Change of life and its neurosis ... 337
Experience with antifebrin ... 338
Cascara Sagrada in Rheumatism ... 339
Summer complaint ... 340
Creasote in Phthisis ... 341
Treatment of the umbilical cord ... 342
Dyspepsia ... 343
Sulphonal; veratrum in pneumonia of children ... 344
Intubation; the treatment of migraine with Indian hemp ... 345
Antipyrin and spirit of nitrous ether; the treatment of gall stones ... 346
Use of chloroform in tracheotomy of children attacked with croup; the contagiousness of cancer; sextuple ... 347
Tyrotoxicon, and its relations to diarrhœa in children ... 348
Cytisin in migraine; goitre can be cured ... 349
The relative value of the bromides; oil of corn; action of boiling water on typhoid bacilli; a cure for drunkenness ... 350

SCIENTIFIC ITEMS.

The home of the great auk; "self-mending snakes" ... 351
Ice on mars; the mosquito a blessing to man; Twins, one white and one black; a sympathetic heart ... 352

PRACTICAL NOTES AND FORMULÆ.

For distressing cough in phthisis; dyspepsia, with chronic constipation and sour stomach; ring worm; simple treatment of itch; treatment of meningetis in children ... 353
Hemorrhages of the lungs and all dangerous internal hemorrhages; incontinence of urine in children; H. C. Woods on colds; typhoid fever ... 354

EDITORIALS AND MISCELLANEOUS.

Bovinine; the Ivy Street Hospital; Mississippi Valley Medical Association; dental instruction; the aunnal meeting of the Southern Surgical and Gynecological Association ... 355
Yellow fever microbe ... 356
Books and pamphlets received ... 357
To medical students ... 359
Special notes ... 360

